# Perceived Health Impacts of Surface Mining: Local Perspectives from the Mining Communities in Libjo, Dinagat Islands, Philippines

**DOI:** 10.3390/ijerph22030365

**Published:** 2025-03-02

**Authors:** Jessa Mae T. Lazarte, Connie Fern Miranda, Ruel S. Apas, Eulogio S. Auxtero, Angeline M. Atacador, Marco Laurence M. Budlayan, Ferdinand Michael B. Calo, Rey Marc T. Cumba, Gladys Edilo, Jade C. Jusoy, Felmer S. Latayada, Ronieto Mendoza, Edmar G. Pantohan, Temmy P. Vales, Mark Vincent Vergara, Joey Arles O. Vergara

**Affiliations:** 1 Department of Physics, College of Mathematics and Natural Sciences, Caraga State University, Ampayon, Butuan City 8600, Philippines; rsapas@carsu.edu.ph (R.S.A.); esauxtero@carsu.edu.ph (E.S.A.J.); amatacador@carsu.edu.ph (A.M.A.); mmbudlayan@carsu.edu.ph (M.L.M.B.); fmbcalo@carsu.edu.ph (F.M.B.C.J.); rtcumba@carsu.edu.ph (R.M.T.C.); ggedilo@carsu.edu.ph (G.E.); e.g.pantohan@gmail.com (E.G.P.); jovergara@carsu.edu.ph (J.A.O.V.); 2 Department of Sociology, College of Humanities and Social Sciences, Caraga State University, Ampayon, Butuan City 8600, Philippines; cbmiranda@carsu.edu.ph; 3 Department of Chemistry, College of Mathematics and Natural Sciences, Caraga State University, Ampayon, Butuan City 8600, Philippines; fslatayada@carsu.edu.ph (F.S.L.); tpvales@carsu.edu.ph (T.P.V.); 4 Department of Electronics Engineering, College of Engineering and Geosciences, Caraga State University, Ampayon, Butuan City 8600, Philippines; rnmendoza@carsu.edu.ph; 5 Department of Communications and Humanities, College of Humanities and Social Sciences, Caraga State University, Ampayon, Butuan City 8600, Philippines; mgvergara@carsu.edu.ph

**Keywords:** Libjo, health impact, health determinant, surface mining, health service delivery, qualitative research

## Abstract

This study examines the perceived health impacts of a mining company on residents of Libjo, Dinagat Islands, Philippines, addressing a critical research gap in understanding the perceived health impacts of mining activities in underrepresented regions. The perceived health impacts of mining operations were examined using a qualitative research approach, employing semi-structured interviews. The interview instrument was developed to investigate how mining company activities impact key health determinants, such as water quality, sanitation, income, employment, infrastructure, education, and women’s empowerment. The study was conducted across multiple sites at different distances from the mining area to further investigate the possible distance-based variations. The findings indicate that the mining company has positively impacted several areas, particularly income, employment, infrastructure, education, and women’s empowerment. However, there were mixed perceptions regarding water quality and sanitation. The majority of respondents, particularly those who are near the mining sites, perceived a significant positive impact on health service delivery, attributing it to the company’s support initiatives. Despite these positive impacts, the community expressed concerns about potential negative environmental consequences, such as air and water pollution, deforestation, land degradation, and noise pollution. This study reveals the complex relationship between mining operations and community health, emphasizing the importance of balancing economic benefits and environmental safety. The study further contributes to a deeper understanding of how mining operations affect the community’s health and well-being and their potential distance-based variations.

## 1. Introduction

Mining industries play a crucial role in economic growth and resource extraction [1,2]. However, key mining operations such as drilling, excavating, blasting, crushing, grinding, smelting, refining, and transporting materials [3,4] significantly contribute to the release of pollutants into the environment [5]. For instance, airborne pollutants (particulate matter) generated by mining activities can degrade ambient air quality in the vicinity of mining operations and nearby communities. Numerous studies have linked PM exposure to negative health outcomes, with strong correlations found between long-term exposure to PM and increased mortality from cardiovascular and respiratory diseases [6,7]. This suggests that communities near mining operations may experience a higher number of health issues related to air pollution.

Numerous adverse health effects, including respiratory diseases, lung cancer, tuberculosis, hearing loss, and skin conditions, have been documented in both mining communities and nearby residents [8,9,10,11,12,13]. Among these, respiratory issues and water-related diseases caused by poor air quality and contaminated water are prevalent, with both short- and long-term harmful effects impacting both mining workers and residents, regardless of age or gender. Beyond health concerns, high levels of environmental pollutants also harm crops by damaging their biological processes [13], while sedimentation and degraded water quality ultimately affect fishing and drinking resources [14]. Given the challenges of dust suppression mechanisms and water treatment in terms of operational cost and scalability, a practical, immediate measure is to regularly monitor the affected resources and track health complaints from workers and residents through regular surveys and interviews. This approach provides a more feasible way to address health risks while gaining insights into the environmental impact perceived and experienced by the community [15,16]. However, the primary weakness of community-based monitoring in addressing health hazards is that it requires considerable manpower for data gathering and technical expertise in accurate collection and analysis. Additionally, the success of such programs relies heavily on community engagement and participation, which can be inconsistent due to factors like lack of awareness or motivation [17]. It is therefore important to implement a data collection strategy that is simple, well-guided, and engaging for the participants.

Monitoring of public health through guided questions has been an effective method to capture firsthand information and perception of the community [18,19]. A qualitative approach with a structured or semi-structured interview is an excellent tool for capturing perceptions due to its flexibility, depth, and reliability in capturing qualitative data [20]. A semi-structured interview, in particular, allows for flexible probing based on the respondents’ answers to guide questions [21]. This offers wider room for exploration in the depth of the participants’ perceptions, opinions, emotions, and attitudes while allowing the researchers to set the premise of the conversation, thereby maintaining the focus on the research objectives and consistency across interviews. A semi-structured interview also captures the context behind perceptions in an open and conversational manner, making it particularly useful for understanding complex or sensitive topics involving the community, environment, and public health relevant to this study.

Libjo, a coastal municipality in the Dinagat Islands, Philippines, spans 4226.27 hectares of mining area, primarily producing nickel, with over 253,403 dry metric tons valued at Php 359,273,954 as of 2020 [21]. Home to a population exceeding 18,051, the municipality’s residents largely depend on farming, fishing, and mining for their livelihoods [22,23]. The mining company’s operational term based on the contract began in 2007 and is projected to conclude in 2032. Despite its significant contribution to the mining sector, Libjo’s isolated geographical location has led to the public health challenges faced by its residents being overlooked, leaving critical issues related to their well-being underexplored.

Globally, mining operations in resource-rich but geographically remote areas often raise significant public health and environmental concerns, particularly for host communities [24]. However, localized contexts like Libjo remain underrepresented in the literature, limiting understanding of how such industries shape health determinants, including water quality, sanitation, income, employment, and overall quality of life. Importantly, the unique perspectives of individuals and communities directly impacted by mining operations have yet to be systematically documented or analyzed in this region. Moreover, the distance-based variations of the perceptions in these remote mining locations are often overlooked. This lack of attention highlights a pressing research gap: the need to examine and understand the nuanced, multifaceted health impacts of mining activities on vulnerable populations in isolated areas and to explore how this perception may vary with proximity to the mining site. By addressing this gap, this study aims to provide globally relevant insights into the complex interplay between economic development, environmental sustainability, geographical location, and community health, offering a valuable contribution to both local and international discussions on responsible mining and its implications for human welfare.

In this study, we report the perceived health effects of the residents towards the mining activity in Libjo, Dinagat Islands, and its nearby area as a host community of a mining company. We focused on the perceived health effects of the identified five significant locations relative to the mining site, namely San Antonio (Balite and Melody), General Aguinaldo, Navarro, and Malinao. The respondents working at the mining site were also included. We captured the health impacts perceived by the residents through a guided interview with an instrument adopted from Himmelsbach et al. [25]. Key health determinants directly related to the perceived impact, such as drinking water, sanitation, and access to healthcare and services, were captured. Determinants such as road networks, electricity, employment, and education were also included in the survey. These factors are considered important health determinants because they collectively influence the overall well-being of individuals and communities. Access to clean drinking water, adequate sanitation, and healthcare services directly impacts disease prevention, treatment, and general health outcomes [26,27]. Meanwhile, broader determinants like road networks, electricity, employment, and education shape the social and economic environment, enabling better access to resources, opportunities, and infrastructure that support healthy living [28]. Together, these factors play a critical role in shaping both immediate health outcomes and long-term quality of life. These health determinants were also used by other studies in capturing public health perception [25,29].

The study is structured around the framework of sustainable development, which emphasizes the need to balance environmental, social, and economic factors to achieve true sustainability [30,31]. In the context of mining, the social dimension plays a crucial role, as it directly affects community well-being, public health, and socio-economic stability [32]. Understanding how communities perceive health is vital for assessing the broader social impacts of mining, particularly in relation to quality of life and well-being [33]. This study explores how individuals in mining-affected areas perceive health risks and well-being, focusing on indicators such as self-reported health status, healthcare access, and environmental exposure [34]. By analyzing these perceptions, the research aims to contribute to a deeper understanding of sustainable development in mining contexts. Specifically, the study seeks to answer two key questions: (i) What health impacts do mining communities perceive? and (ii) How do these perceptions vary across different areas?

## 2. Materials and Methods

### 2.1. Study Sites

**Figure 1:**
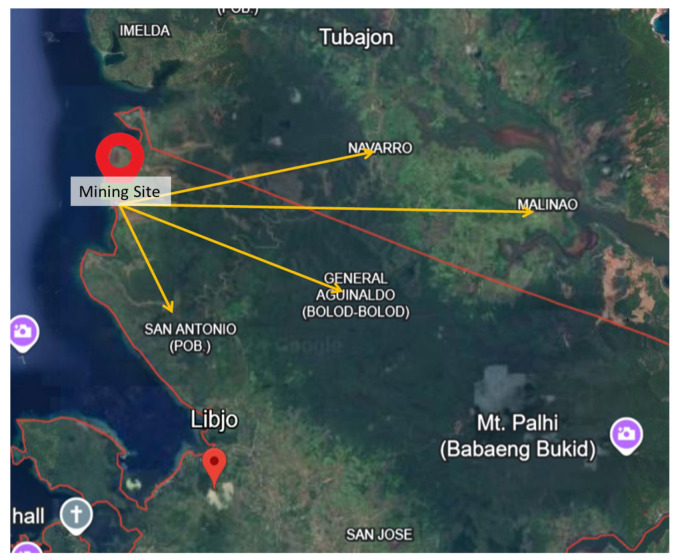
Portion of the map of the province of Dinagat Islands and the locations of the five host communities relative to the nickel surface mining site in Libjo [24].

For the study presented, key informant interviews (KIIs) were conducted at the nickel surface mining site and five surrounding communities in the municipalities of Libjo and Tubajon, Province of Dinagat Islands, Philippines. Key informants were identified from the five communities—Brgy. Malinao and Brgy. Navarro in the Municipality of Tubajon, and Brgy. General Aguinaldo and Brgy. San Antonio (Sitio Balite and Sitio Melody) in the Municipality of Libjo—and among the employees at the mining site as shown in Figure 1. Respondents are identified accordingly, and their roles in the community are to explore variations in perceptions of health impacts caused by mining operations.

### 2.2. Sampling and Recruitment of Study Participants

Regular communication among the project team, the mining company management officials, and stakeholders was secured during the project implementation. An initial site visit was conducted to assess the extent of the effects of nickel surface mining operations in the area. The project team conducted a KII along with the designated Community Relations Officers (CRO) and barangay officials. The CROs are representatives from the mining company who work with the community officials to ensure the implementation of the programs pledged by the company for the community.

Research participant selection was conducted by purposive sampling of the major stakeholders, which included mining officials and community leaders. Respondents were of various genders and legal ages and were required to have resided or served in the community for at least six months. The targeted sample size was 3–4 people per stakeholder group, adding up to 10 people per study site. Thus, the overall targeted sample size was at least 50 respondents across identified communities. Previous studies in rural areas successfully used transect walks to systematically identify relevant infrastructures and services for local communities and establish a first relationship with residents and the study area [25,35,36]. A more detailed description of the transect walk methodology is provided elsewhere [25].

### 2.3. Data Collection and Instrument

Data were collected in the third week of May 2024, but preparations for the fieldwork started before arrival in the field. Regular debriefings were conducted before the KIIs to ensure a shared understanding of project objectives and data collection methods. This helped standardize data collection practices and yielded more reliable and comparable results.

The project team facilitated the KIIs along with the CROs and local officials. A semi-structured interview guide adapted from the study of Himmelsbach et al. served as the basis for the interviews [23]. The first part of the survey questionnaire was the informed consent that orients the respondent to the purpose of the study, risks and benefits, voluntary participation, and privacy and confidentiality.

The KII comprised two parts. Part I collected the background information of the respondents. Meanwhile, Part II comprised open-ended questions to explore the health impacts of the mining operations in the community and the health system response to these impacts as perceived by the three stakeholder groups (i.e., mining officials, healthcare providers, and community leaders). Part II required the interviewees to recognize the factors concerning health and health service delivery. The first question asked the participants to identify the general health impacts of mining in their community. The perceived general impact of mining on public health was explored by the qualitative categorization: “strongly positive”, “a bit positive”, “no difference”, “a bit negative”, and “very negative”. The perceived benefits in health services delivery experienced by the community due to the mining company was also explored using the descriptions: “strongly beneficial”, “a bit beneficial”, and “no benefit”. Subsequently, to further provide context for the perception with regard to these categories, the participants were asked to rate selected health determinants that are commonly affected by mining as having “improved”, “had no effect”, or “worsened.” Based on previous research studying how mining operations affect community health [25,36,37,38], the selected health determinants were “drinking water”, “sanitation”, “income levels”, “employment”, “road networks”, “electricity”, “educational institutions”, and “women’s empowerment”. Note that the respondents answered verbally and were given enough freedom and time to provide reason and context for their answers. The KIIs were carried out at the interviewees’ offices or a location where adequate privacy and minimal distraction were assured. The KIIs were conducted using their local language (Bisaya/Surigaonon). The project team was responsible for documenting the interviews using a voice recorder or taking notes. After the KIIs, debriefing sessions were also conducted among the project team, CROs, and supporting locals to discuss the progress of data collection and evaluate important findings.

### 2.4. Quality Assurance

Prior seminars and training of the interviewers guaranteed the quality of the collected data from the field. Furthermore, the debriefings have proven helpful in ensuring a harmonized data collection and that all interviewers understand the perceived impacts in the study area [37,39].

### 2.5. Data Management and Analysis

The qualitative data collected were analyzed using a combination of categorical response analysis and thematic analysis. Percentages were calculated for categorical responses to each question to provide an overview of response distribution. Additionally, translated excerpts from participant interviews were included to offer contextual depth and illustrate key perspectives. To systematically analyze participants’ perceptions of observed health impacts, the researchers employed pattern coding techniques. Through this process, five overarching themes emerged: (1) air quality, (2) water quality, (3) deforestation, (4) land degradation, and (5) noise pollution. These themes were identified based on recurring patterns in participant responses, ensuring a structured and meaningful interpretation of the data.

Substantial response variations were further analyzed by reviewing the KIIs. In line with the project’s objectives, the analysis aims to capture the range of perceptions of health impact among different actors and communities (host and neighboring). The whole data analysis is continuous; transcripts were reviewed several times to verify responses and re-examine KIIs. Excerpts from the interviews were used to highlight the key findings. These excerpts were further translated and transcribed from Bisaya/Surigaonon to English, ensuring that they reflected the exact statement made in the original local language.

### 2.6. Ethical Considerations

The researchers sought consent from the office of the Punong Barangay (local government unit) prior to conducting the study. They were informed beforehand that KIIs would be undertaken to generate perceptions on the health impacts of the nickel surface mining site in their locality. During the KII sessions, the researchers explained the objectives and procedures of the study and provided a written informed consent form. Participants were assured of voluntary participation, the absence of risks, and the lack of compensation. The analysis of data ensured anonymity and confidentiality. Transcripts and other data were uploaded and stored in a data management system accessible only by the research team.

## 3. Results

### 3.1. Study Population and Demographics

A total of 55 study participants were interviewed at the surface mining sites and their surrounding communities. The respondents ranged in age from 21 to 67 years old and had resided in the community for at least six months. A total of 57.4% of the respondents had resided in the community for more than 20 years, 3.7% between 16 and 20 years, 14.8% between 11 and 14 years, 7.4% between 6 and 10 years, 11.1% between 1 and 5 years, and 5.6% for less than a year. This suggests that most participants had knowledge of the conditions of the community that existed before and after establishment of a mining company. In terms of occupation, the respondents included housewives, farmers, fisher folks, teachers, social workers, carpenters, park rangers, utilities workers, child development workers, environmental foremen, businessmen, vendors, community relation officers, nurse aids, engineers, clerks, haulers, barangay officials, barangay nutrition scholars, barangay health workers, and mining workers. A detailed distribution of the study participants is presented in Table 1.

### 3.2. Perceived Impacts of Mining on Public Health Based on the Geographical Location

**Figure 2:**
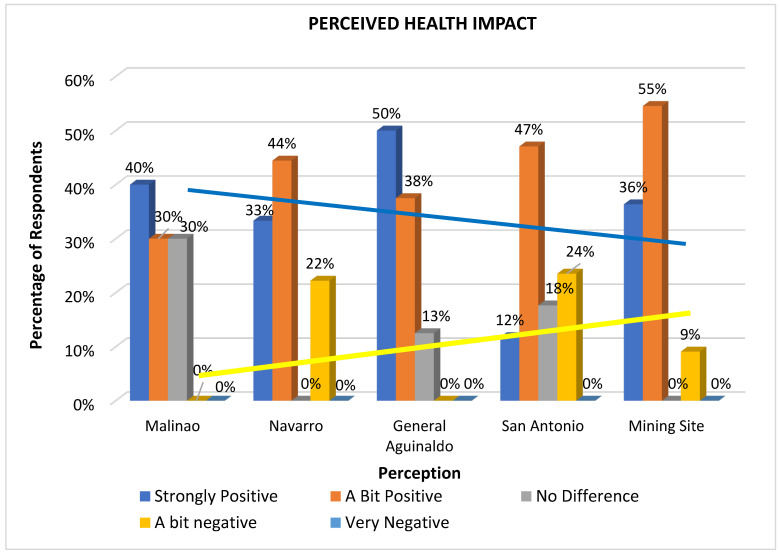
Perceived health impacts of nickel surface mining operations across five affected communities in Dinagat Island.

Figure 2 shows the perceived general health impact percentages across five mining-affected areas: Malinao, Navarro, General Aguinaldo, San Antonio (Balite and Melody), and mining sites. The areas are arranged from the farthest, Malinao, to the closest, San Antonio, in relation to the nickel mining consolidation area/operations, along with the mining site itself.

In the farthest areas (Malinao, Navarro, and General Aguinaldo), approximately 10% to 30% of respondents believe there is no difference in public health with or without mining operations. Between 30% and 50% perceive a slightly to strongly positive impact, while only 0% to 22% perceive negative impacts, with Navarro having the highest at 22% of respondents saying there is a “bit negative” impact.

In contrast, in the closest area (San Antonio, excluding mining site employees), only 12% of respondents view the health impact as strongly positive, though 47% see it as a bit positive. Additionally, 24% of respondents in these areas report a “bit negative” impact and 18% report no difference, suggesting that the perception is mixed and varied near the mining operations.

Among mining site respondents, 38% report a strong positive health impact, 52% see a “bit positive” impact, and surprisingly, 10% perceive a slight negative impact.

The yellow linear trend line indicates that perceived health impacts become less positive as the distance to the mining site decreases. This finding aligns with the fact that the nickel mining operations are located in San Antonio and at the center of the mining site. These results, suggesting declining positive health impacts of nickel surface mining operations in Libjo, lead to several possible implications: (1) insufficient health support in terms of medical check-ups and the provision of essential medicines, (2) less effective dust suppression mechanisms, which may cause respiratory issues, (3) less aggressive environmental rehabilitation efforts, and (4) inadequate siltation ponds, which may cause sediments accumulations to nearby bodies of water.

### 3.3. Perceived Impacts of Mining on Health Determinants

The perceived impacts of mining on population health have been analyzed through several key health determinants: drinking water, sanitation, road infrastructure, employment, income levels, electricity, educational institutions, and women’s empowerment. Health determinants like drinking water quality and sanitation directly reflect the impact of mining activities on public health. On the other hand, factors such as road networks, employment, income levels, electricity, educational institutions, and women’s empowerment influence health awareness, the availability of healthcare facilities, and the delivery of health services. These determinants offer a broader understanding of how mining indirectly affects community well-being.

**Figure 3:**
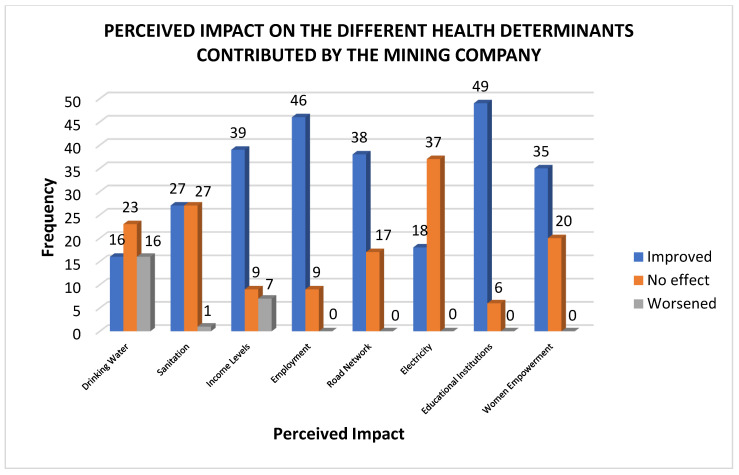
Perceived impacts of mining on population health through several key health determinants.

As shown in Figure 3, the majority of the respondents observed no significant change in the quality of the drinking water. Intriguingly, an equal response was recorded for worsened and improved drinking water quality. The split between improvement and worsening suggests a mixed impact of mining on water quality. Mining activities can affect water resources through contamination. Still, some respondents may have seen improvements due to mitigation efforts by the mining company, such as providing drinking water, water tanks and containers, and other alternative water sources. The survey results on sanitation show an equal response between those reporting no effect and those indicating improvement, suggesting a complex and mixed situation in the community. The split in perspectives could point to disparities in access to sanitation services offered by the community across different locations. This captures the residents’ statements that sanitary product distribution is unequal across the community. Road networks, employment, income levels, educational institutions, and women’s empowerment were perceived to undergo a collective improvement with the mining company’s presence, as evidenced by the dominant “improved” response across the area in the mentioned indicators. On the other hand, development in electricity seemed to be stagnant, with a collective “no effect” response and no “worsened” responses across the area.

#### 3.3.1. Drinking Water

**Figure 4:**
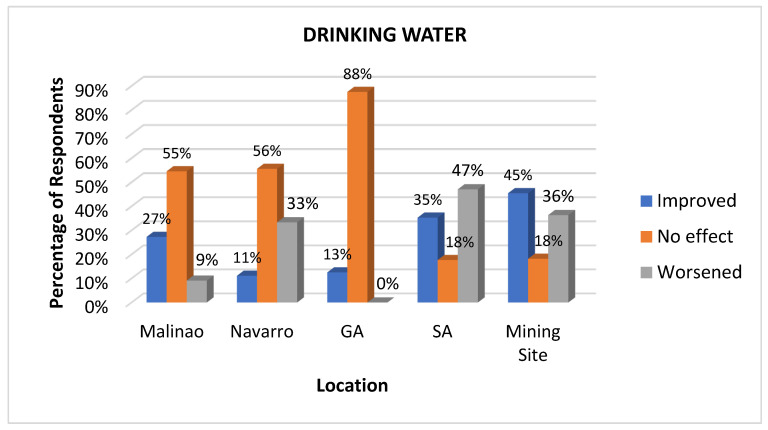
Impacts of mining on drinking water quality as perceived at various sites.

The impact of the mining activity on the water quality as perceived by the residents varies by location, as presented in Figure 4. Drinking water is an important health determinant because it is essential for maintaining life and overall health. It plays a critical role in various physiological processes and waste elimination. Access to clean and safe drinking water prevents waterborne diseases such as cholera, typhoid, and diarrhea, which may cause morbidity and mortality in the community, especially in its vulnerable populations [26,40]. Interestingly, more than 50% of respondents in three locations, namely Malinao, Navarro, and General Aguinaldo, responded with “no effect” with respect to drinking water quality, with General Aguinaldo giving the highest level of this response at 88%. The general “no effect” response from these areas could be due to their geographical locations being far from the mining site.

In the same barangay, respondents claimed that the mining company built a water reservoir near the area, but it was not operational; hence, there was no perceived improvement in the drinking water supply. Meanwhile, San Antonio, located near the mining area, reported a significant decline in drinking water quality, with a total “worsened” response of 47%. According to the residents, the worsened drinking water quality is associated with the mining operations near the watershed, which further causes sedimentation in the immediate area. Specifically, the residents who are dependent on springs for their drinking water suffered from worsened drinking water quality. Meanwhile, the majority of the mining workers responded with an “improved” response (45%) due to the provision of drinking water facilities within the mining site. They mentioned that the mining company provided workers with tanks for mineral drinking water. However, it is worth mentioning that a significant “worsened” response (36%) was recorded among the respondents from the mining area. This response is attributed to the observed turbidity in the natural drinking water available within the vicinity. One resident from the community stated that:

*“Sa tubod mi naga-inom sauna, karon dili na mainom... gikalibanga na”. (We used to drink water from the spring before, but now it’s no longer drinkable... it causes diarrhea.) (Respondent—Barangay Health Worker).*

#### 3.3.2. Sanitation

**Figure 5:**
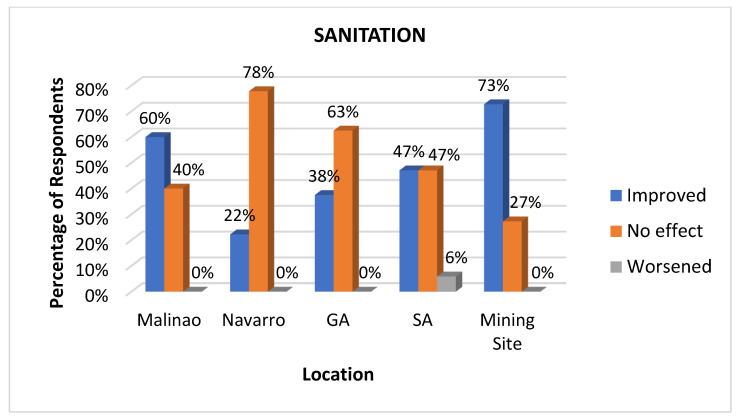
Impacts of mining on sanitation as perceived at various sites.

Sanitation refers to the practices designed to promote hygiene by safely managing human waste, providing a clean water supply, and ensuring proper waste disposal. These measures are conducted by the community to maintain hygienic conditions for public health. This is a vital health determinant because it directly impacts hygiene and the prevention of disease. Proper sanitation systems, including access to clean toilets and proper waste disposal, reduce the risk of waterborne illnesses, which are major public health concerns, especially in underserved areas like Libjo [41]. By looking at sanitation as a health determinant, communities experience better health outcomes, making it an essential component of sustainable development and public health strategies. In the context of this research, we emphasized sanitation based on the functionalities of the toilets and waste disposal mechanisms in the community. There was a diverse response, addressing issues such as toilet functionality, waste disposal, and perceptions of hygiene improvements, as seen in Figure 5. The percentages of residents from Malinao and of the mining site workers responding that sanitation was “improved” were 60% and 73%, respectively. This perceived improvement in sanitation is associated with the provision of toilet bowls and construction materials for toilets in the mentioned area.

*“Nanghatag ug toilet bowl and materials ang mining company.” (The mining company distributed toilet bowls and materials.) (Respondent—Barangay Official)*

On the other hand, two locations, namely Navarro and General Aguinaldo, generally responded that there was no improvement in this indicator. In contrast, San Antonio responded with an equal percentage of “improved” and “no improvement”. Moreover, it is worth noting that the “worsened” response is generally low across the surveyed area (0–6%), which suggests that the mining company’s provision of toilets and sanitary materials helped improve sanitation across the communities.

#### 3.3.3. Road Network

**Figure 6:**
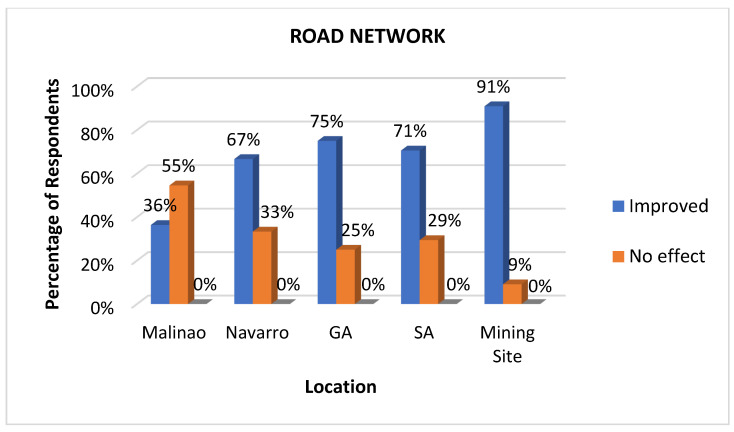
Impacts of mining on road networks as perceived at various sites.

Road networks are an important health determinant because they facilitate access to essential healthcare services, emergency care, and resources needed for well-being. Reliable road infrastructure ensures that residents can reach hospitals, clinics, and other health facilities easily [42]. Additionally, road networks support the delivery of goods, such as clean water, food, and medicines, while enabling economic opportunities that indirectly contribute to better health outcomes through improved living standards [43]. This work revealed that as the geographical location draws nearer to the mining site, an increasing trend in the perceived improvement of the road network among the identified locations with the assistance of the mining company is agreed upon by the respondents. As observed in Figure 6, in four locations, namely Navarro, General Aguinaldo, San Antonio, and the mining site, the results show that the majority of the residents agree that there is an improved road network in their area. One respondent stated that:

*“Naghimo ug agianan para sa mga estudyante ang kumpanya.” (The company built a pathway and road for the students.) (Respondent—Fisherman)*

This includes the construction of concrete roads, farm-to-market roads, and road widening with the aid of the nickel mining company. Meanwhile, Malinao showed no significant improvement in its road network, probably due to its location farthest from the nickel mining company. Some agreed that road network improvements exist in their area, but these improvements are not merely due to the identified nickel surface mining company. Other mining companies near Malinao have also participated in improving the road network in the area.

#### 3.3.4. Employment

**Figure 7:**
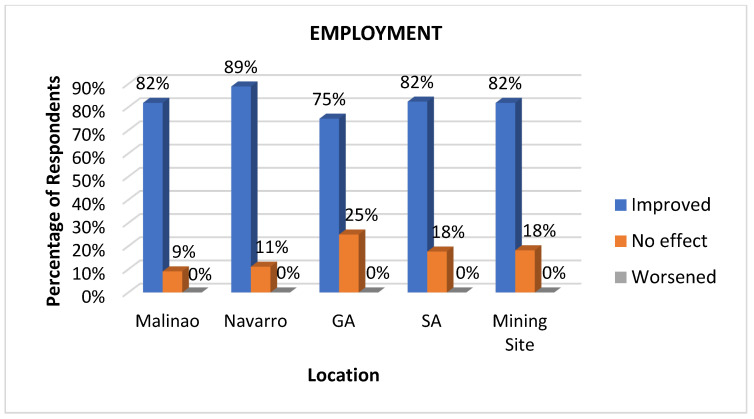
Impacts of mining on employment as perceived at various sites.

Employment is a critical health determinant because it provides individuals with financial stability to access essential resources such as nutritious food, safe housing, and healthcare services [44]. Having a job also offers a sense of purpose and social connection, which positively impacts mental well-being and reduces stress. Furthermore, employment opportunities contribute to broader economic development, helping to reduce poverty and health disparities within communities [45]. As shown in Figure 7, the employment rate in all locations is relatively high. This highlights the positive contribution of the mining company in providing employment opportunities for local residents. According to respondents, the company prioritizes hiring community members, provided that they meet the job qualifications. Residents were employed in various roles, such as drivers, utility workers, clerks, and cooks. Additionally, the company offered pump boat rides that improved residents’ access to jobs and livelihoods. The company also initiated livelihood projects for the community through its Social Development and Management Program (SDMP), further supporting employment and economic growth.

*“Nakasulod sa kumpanya ug trabaho unya nakalibre ug pumpboat sa trabaho ang mga tao.” (People got a job at the company and were given a free pump boat for work.) (Respondent—Fisherman)*

#### 3.3.5. Income Levels

**Figure 8:**
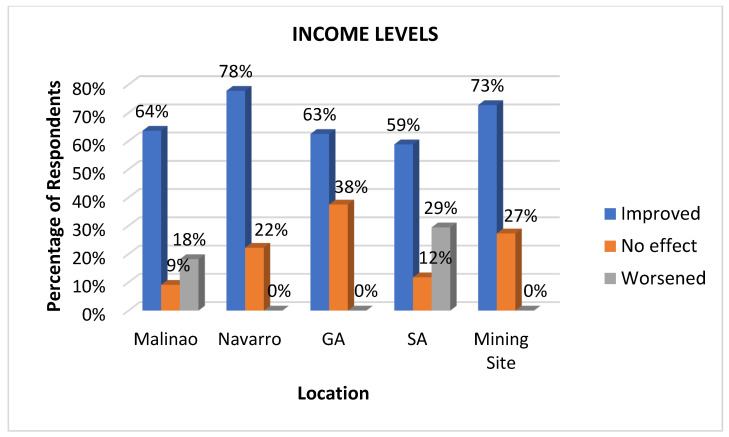
Impacts of mining on income levels as perceived at various sites.

Income levels are an important health determinant because they directly influence an individual’s ability to afford necessities like nutritious food, safe housing, and quality healthcare [46]. Higher income levels are often associated with better access to education, resources, and opportunities that promote healthy living. Conversely, low income can lead to financial stress, limited access to healthcare, and increased exposure to environmental and social risks, all of which contribute to poorer health outcomes [47]. Parallel to the perceived impact on employment, a significant improvement in income level was generally observed, as presented in Figure 8. The highest percentage of respondents who reported improved income levels were from Navarro (78%) and mining sites (73%). General Aguinaldo (69%) and Malinaso (64%) also show a significant number of people with improved income, while San Antonio (59%) has the lowest percentage of perceived income improvement, though still a majority. On the other hand, San Antonio (29%) has the highest percentage of respondents who saw no effect on their income, followed by GA (25%). In comparison, Malinao (18%) and Navarro (19%) have similar levels of “no effect” responses. In terms of worsened income levels, Malinao and San Antonio perceived higher and worsened incomes. The adverse impact on income levels is associated with inconsistent operations during the off-season and salary delays. These results may suggest that respondents in certain areas, particularly those in Navarro and the mining site workers, benefit more positively in terms of income level due to the mining company. In contrast, others, such as those in Malinao and San Antonio, are more adversely affected or experiencing stagnation.

#### 3.3.6. Electricity

**Figure 9:**
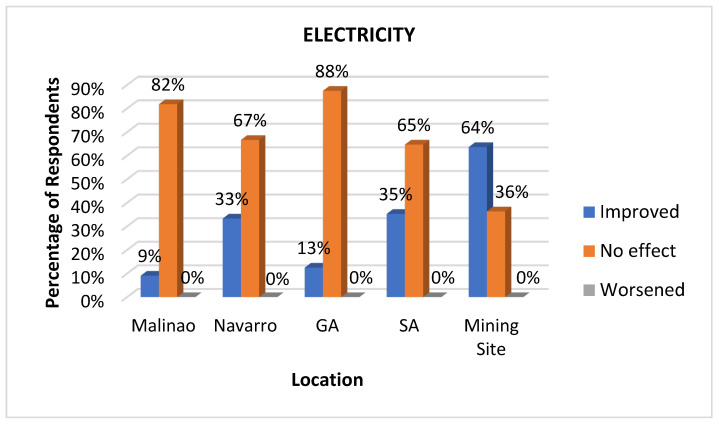
Impacts of mining on electricity as perceived at various sites.

Electricity is an important indicator of health impact because it directly influences the delivery of health services and the functionality of health facilities catering to individual and public health. It is a crucial determinant of health because it also supports the infrastructure required for clean water, food safety, environmental quality, and overall well-being. A lack of access to reliable electricity can result in a deterioration of public health and limited access to essential healthcare services, particularly in rural areas. As anticipated, the mining site workers (64%) had the highest proportion of respondents reporting improved electricity services (Figure 9). Interestingly, the majority of respondents across the area experienced no change in their electricity service, with General Aguinaldo (88%) and Malinao (82%) showing the highest percentages of respondents reporting no effect. Navarro (67%) and SA (65%) also held significantly large majorities stating no change. None of the locations reported a significant worsening of electricity services, with all categories showing 0% except Malinao, where 9% of respondents said that their electricity services had worsened.

#### 3.3.7. Educational Institutions

**Figure 10:**
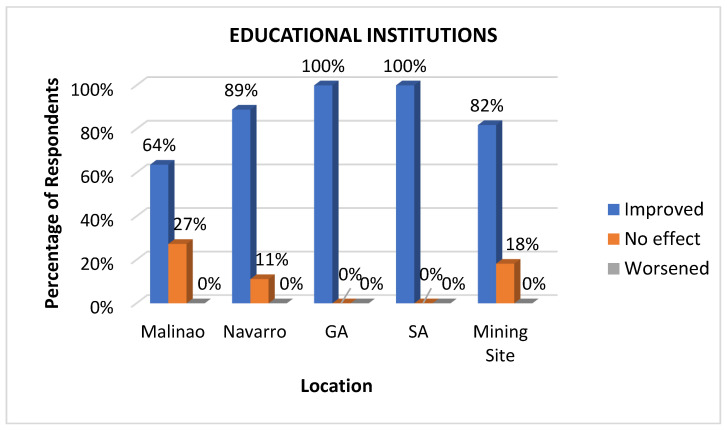
Impacts of mining on educational institutions as perceived at various sites.

Educational institutions constitute an important health determinant because they provide knowledge and skills that empower individuals to make informed health decisions, such as practicing good hygiene, maintaining proper nutrition, and seeking timely medical care [48]. Education also contributes to better employment opportunities, leading to improved socio-economic conditions that support overall well-being. From another perspective, schools often serve as platforms for health interventions, including immunizations, health screenings, and the promotion of healthy behaviors, making them integral to fostering healthier communities; hence, considering educational institutions as health institutions is necessary [49]. Respondents from various communities acknowledged the positive influence and enhanced impact of mining on educational institutions. The most significant improvements were observed in General Aguinaldo (100%) and San Antonio (100%), followed by Navarro (89%), the mining site (82%), and Malinao (64%), as exhibited in Figure 10. These advancements were largely attributed to the mining company’s initiatives, which included constructing or refurbishing school buildings, offering scholarships, providing subsidies for teachers, donating ICT equipment and school and office supplies, installing water systems, and supplying uniforms, school health services, and feeding programs.

*“Naa gihatag school materials and budget for school renovation, repair ug building.” (School materials, a budget for school renovation and repair, and a building donation were provided.)*

#### 3.3.8. Women’s Empowerment

**Figure 11:**
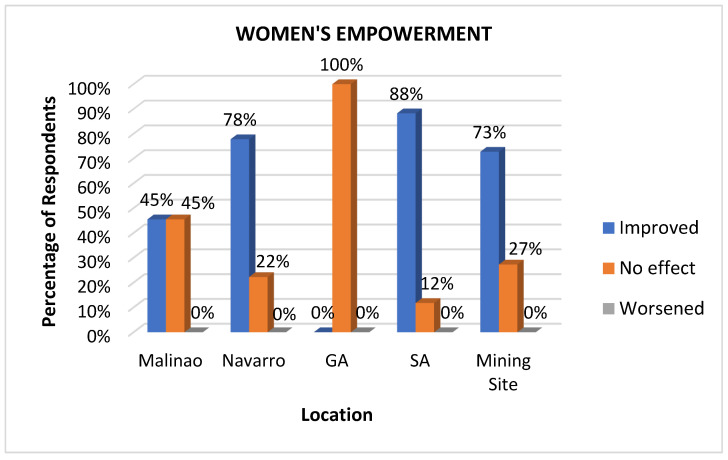
Impacts of mining on women’s empowerment as perceived at various sites.

Women’s empowerment is a vital health determinant because it enables women to make informed decisions about their health, including reproductive health, nutrition, and access to healthcare services [50]. In the local context, where women often manage households and care for children, empowering them enables greater investment in their families’ well-being, resulting in healthier children and stronger communities [51]. Additionally, promoting gender equality and providing women with education, economic opportunities, and social support reduces health disparities and improves overall public health outcomes. Significant improvement responses in women’s empowerment were observed in San Antonio (90%) and the mining site (75%), as revealed in Figure 11. This significant improvement is attributed to the availability of monthly health check-ups and immunizations for pregnant women and the provision of women’s hygiene and sanitary kits. One respondent from Brgy San Antonio stated:

*“Sa pagsugod sa mining company, naay mga programa para sa mga babae nga livelihood sama sa paghimo ug asin, cassava cake ug pag atiman ug poultry... kung mangayo pud, naay tambal nga ginahatag ang kompanya…” (Since the mining company started, there are programs for women’s livelihoods such as salt-making, cassava cake production, and poultry care. When we request, the company also provides medicine to us...)*

Livelihood programs offered by the mining company specifically for women, such as the poultry farms, cassava cake cooking, and salt-making businesses mentioned in the quotation, add to the perceived improvements. Moderate improvements were observed from Malinao (50%) and Navarro (50%), while General Aguinaldo showed minimal improvement, with nearly no respondents reporting progress in women’s empowerment. Interestingly, 100% of respondents from General Aguinaldo, situated in the middle of the surveyed sites, reported no change in women’s empowerment, suggesting no significant initiatives, interventions, or impacts in this area. Navarro (50%) and Malinao (50%) also show a significant proportion of respondents reporting no change. Minimal to no worsened responses were observed across the area, indicating no noticeable regression in women’s empowerment efforts.

### 3.4. Perceived Impact of Mining Company on Health Service Delivery

**Figure 12:**
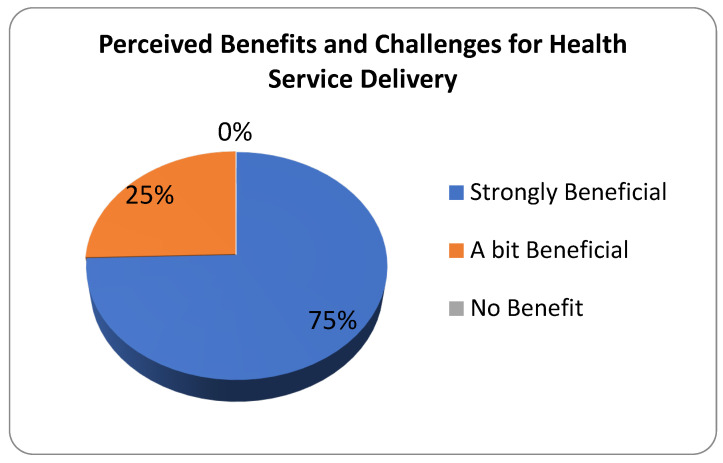
Perceived impacts of mining on overall health service delivery.

The general impact of the nickel mining company on healthcare delivery was also assessed using the following indicators: service availability, general service readiness, and service-specific readiness (Figure 12). Service availability includes health infrastructure (e.g., health center improvement), health workforce (additional nurse/doctor), and service utilization (e.g., check-ups, medical missions). General service readiness includes basic amenities (e.g., health and wellness or recreation), basic equipment (e.g., thermometer, BP apparatus, stethoscope), precautions for infection prevention (e.g., vitamins, deworming, feeding program), diagnostic capacity, and essential medicines. Service-specific readiness includes the following: care related to maternal, child, and adolescent health; malaria diagnosis and treatment; tuberculosis services; all healthcare related to HIV/AIDS; diagnosis and treatment of sexually transmitted infections (STIs); noncommunicable disease diagnosis and management such as diabetes, cardiovascular disease, chronic respiratory disease, and cervical cancer screening; basic and comprehensive surgical care; blood transfusion; and laboratory capacity.

Results show that 0% of the respondents answered, “No benefits”, which suggests that they had an idea of what healthcare services the nickel mining company provides to its employees and host communities. The majority (75%) of the respondents across all identified locations agreed that the health service delivery by the nickel mining company to the community is strongly beneficial. This suggests that the majority have full knowledge of the healthcare services that the company is providing and is a recipient of those services. This positive response is probably due to the following reasons: health centers were improved with the help of the company; basic amenities, sanitary kits, and essential medicines were made available to the host communities; the company gives assistance to medical missions; the company conducts feeding programs; and materials needed for building toilets such as bowls and septic tanks were provided to every household in the host community for free. Community members can also avail themselves of healthcare services such as free check-ups from the company’s healthcare personnel, from whom emergency service is available 24 hours a day. The company also assists in blood transfusions or blood donations upon the request of their employees. The employees also undergo HIV tests and general check-ups annually to ensure that they are fit for work. Furthermore, basic amenities for recreational activities are present and made available to promote physical fitness among the employees and host communities.

The rest of the respondents (25%) expressed that the health services delivered by the nickel mining company to the community are only a bit beneficial. This suggests that some respondents do not have full knowledge but at least an idea of what healthcare services the company is providing, and may not be among the recipients or may not have received 100% support from the company. They may have seen improvements in healthcare facilities, but they may also have noticed that these are insufficient to cater to all.

### 3.5. Identified Themes in Health Perceptions and Challenges

From the conducted interviews, five overarching themes emerged: (1) air quality, (2) water quality, (3) land degradation, (4) deforestation, and (5) noise pollution. These themes were identified based on recurring patterns in participant responses. The most dominant theme was the observed impact of mining activity on air quality, particularly during peak operations. According to the respondents, the air quality was dramatically degraded, especially during peak operation time, due to the emission of smoke and dust from excavation and transportation of mineral-containing soil from the site to the hauling ships. Despite mitigation measures, such as water sprinkling on tracking roads to reduce dust emissions during soil transport, a significant amount of dust still reaches households and farms. While no formal medical diagnoses have been made, locals associate poor air quality with the respiratory issues they experience. Notably, this extreme presence of dust is observed only during active mining operations and primarily affects communities near the mining sites. Furthermore, during off-seasons, air quality improves and returns to normal levels.

*“Ang ubo ug sip-on dili mawala tungod sa abog. Wala ni sauna.” (The cough and colds don’t go away because of the dust. This wasn’t the case before.)*

Another salient theme that emerged from the interviews was the impact of mining activity on water quality. According to the statements gathered, the effect of mining spans from drinking water to seawater. Respondents emphasized that the mining company provides drinking water to the community, as their natural water sources were not potable anymore. Efforts by the mining company to create water reservoirs for the communities were also appreciated, but they were not yet functional as of the time of study. Some residents also expressed worries over the mining company operating near their watershed. Meanwhile, on the coastline located near the mining areas, residents observed sedimentation and run-offs from the mining area and siltation ponds, especially during the rainy season. This consequently affected the residents whose livelihoods are dependent on shallow-sea and inshore fishing.

*“Grabe ang paglubog sa tubig sa baybay ug suba samot na kung mag-ulan.” (There is extreme sedimentation in the river basins and on the shore, especially during rain.)*

One of the prominent themes that emerged from the qualitative analysis was the issue of noise pollution, which was consistently mentioned by respondents near the mining sites. Participants noted that the noise generated by trucks transporting mined soils and other heavy machinery was a constant disturbance, particularly during nighttime. This issue is exacerbated during peak mining seasons, when operations run 24 h a day, disrupting the sleep patterns of local residents. The continuous presence of noise—whether from transport activities or machinery—creates an environment that is not only physically uncomfortable but also has potential long-term impacts on mental health. Chronic sleep disturbances, as cited by participants, can lead to stress, anxiety, and other related health issues, highlighting the broader implications of mining operations for the well-being of affected communities.

*“Noisy ang ear end cover ng dump truck…” (It’s noisy because of the rear end cover of the dump truck…)*

In addition to noise pollution, land degradation and deforestation emerged as significant, though less prominent, concerns. These environmental issues, while often linked to the physical effects of mining, also have indirect public health impacts. Land degradation reduces arable land, leading to food insecurity and socio-economic instability, while deforestation disrupts ecosystems, degrades air quality, and limits resources like fuel and building materials. From a public health perspective, these changes can lead to respiratory issues, disease spread, and lower quality of life. While less frequently mentioned, these environmental concerns are crucial in understanding the broader health impacts of mining, as they influence both resource availability and access to essential services.

## 4. Discussion

The participants across the four host communities have observed health-enhancing impacts from the mining company regarding road networks, employment, income levels, educational institutions, and women’s empowerment. The residents see the mining-associated infrastructure as an opportunity, as it satisfied multiple objectives. Apart from promoting economic activity for the company, the improved road infrastructure offers significant indirect benefits to the host community, which previously relied on pump boats as their primary mode of transportation. The road widening and creation of alternative routes have greatly enhanced accessibility, providing residents with more efficient and safer options for daily travel and facilitating quicker access to essential and/or emergency services, markets, and social opportunities. This result emphasizes how essential an effective transport network is for safe, effective, and smooth mining operations activity (product movement and employee access) and the day-to-day community residents’ travel [52]. On the other hand, poorly maintained roads or those affected by extraction operations may make it more difficult to obtain healthcare and raise the risk of pollution and traffic [53].

Respondents have also observed a positive change in economic activity due to the mining company’s presence. This is seen in the improved employment and income levels due to the direct work opportunities offered within the mining operations and by the Social Development Management Program (SDMP), along with other secondary economic activities. This result corroborates other studies highlighting the significant influence of the mining sector on economic development and growth [2], which in turn allows more resources for social services such as health, education, and welfare, increasing community livability and lifestyle factors [52,53,54,55].

It is worth noting that the participants across areas perceived an improvement in women’s empowerment. Some participants directly benefited from the company’s programs and initiatives, while others experienced indirect advantages, such as improved community resources or economic opportunities, which could positively impact their overall well-being. Initially, participants perceived certain projects as beneficial but later reported sustainability issues that undermined these benefits. This finding highlights the need to incorporate sustainability considerations into the mining company’s impact assessments. While mining companies hold significant potential to drive sustainable development, the risks for local populations, especially women, may outweigh the advantages. This condition emphasizes how crucial inclusive and all-encompassing management techniques are in reducing adverse effects [27,37]. Women’s empowerment is critical to health because it reflects women’s access to healthcare. In particular, the majority of households in the community rely on mothers to nurture the children and the households. Thus, women’s empowerment is tightly connected to mental and physical well-being and family health. It also enables women to take charge of their reproductive health and participate more actively in decisions that impact their families’ and communities’ health.

On the other hand, the results indicate a mixed response to the sanitation improvements in the mining communities. The provision of toilets to the mining communities has improved their sanitation practices. Chen and Zhu [54] found that access to toilets in rural households significantly improved residents’ health. However, some mining communities perceived no effect. This disparity in responses across different locations highlights the complex nature of sanitation improvement. Socio-economic conditions and awareness campaigns may influence how residents perceive sanitation changes.

Most responses indicated no change in electricity services, except for those of mining site workers, who reported improvements. Mining operations rely heavily on electrical systems to support various activities, allowing healthcare services in these areas, like medical facilities, sanitation, and medical devices, to operate efficiently. These improvements could enhance access to healthcare services and positively impact health outcomes, such as more reliable routine care and quicker emergency response times. This is particularly valuable given the mining site’s location on an island with limited healthcare access. Interestingly, the majority of respondents across the area experienced no change in their electricity service. The “no effect” response from mining communities suggests that participants do not attribute the presence of electricity to the mining company, as the national government already provided it before mining operations began in the area. It is important to note that supplying electricity to these communities is the government’s responsibility, not the mining company’s.

In terms of water quality, the perceived impact of mining on water quality varies depending on the location and proximity to the mining site. While most residents in areas farther from the mining activities report no discernible impact on the quality of their potable water, those located closer to the mining site report a substantial decrease in water quality. This implies that residents in close proximity to mining sites frequently express more substantial apprehensions regarding water contamination, which are likely the result of observable changes such as sedimentation or turbidity, which affect critical water sources. In contrast, communities located further away from such activities, where environmental impacts may be less apparent, typically perceive minimal or no impact on water quality. This is corroborated by the findings of Levêque and Burns [55] that there exist differences in water quality perceptions and environmental concerns based on the type of natural resource extraction sites, their proximity, and the density and presence of these sites.

The results emphasize the significant role that mining companies can play in supplementing local healthcare systems, especially in remote areas. The positive response from the respondents suggests that the mining company’s healthcare initiatives have filled a crucial gap in service delivery. Providing general and specific healthcare services, from basic health check-ups to treatment for common diseases, has likely improved the overall well-being of employees and the wider community.

## 5. Conclusions and Recommendations

While the general outlook of the community leans towards positive impact, concerns can be broadly categorized into the following: air quality, water quality, deforestation, land degradation, and noise pollution. Among these, air and water quality are the most affected. In addition, communities located farther from the mining site view the services as less beneficial than those living near the mining site. This implies that the company should reassess the reach and effectiveness of its healthcare programs, particularly in ensuring equitable access. Better communication of available services and addressing any remaining healthcare gaps could enhance the perceived benefits and ensure that all community members feel the positive impact of the company’s health initiatives. Furthermore, collaboration with local health authorities, including the LGU, could help extend the reach of these services and make them more sustainable.

Overall, the findings highlight the need for mining companies to adopt a holistic approach to social development and management programs, one that not only enhances community well-being but also actively engages all stakeholders to ensure inclusive healthcare delivery. Theoretically, this study contributes to the existing literature by bridging the gap in understanding how geographic proximity to mining operations influences community perceptions of health impacts and access to benefits. By providing localized evidence, it enriches the growing discourse on environmental health, emphasizing the interplay between environmental degradation, socio-economic disparities, and health outcomes. These theoretical insights extend current frameworks by integrating proximity-based variations in perceptions and their implications for equitable resource distribution and health service delivery.

Empirically, the research identifies critical areas of concern, such as air and water quality, that demand immediate and targeted intervention. The study’s findings offer detailed, community-specific challenges that policymakers and stakeholders can address through tailored health and environmental policies. This empirical contribution emphasizes the importance of addressing both the direct health impacts and the broader socio-environmental determinants of health, such as infrastructure, education, and access to healthcare, in mining communities.

From a practical perspective, the study offers actionable insights for mining companies and policymakers. Implementing targeted healthcare initiatives that prioritize underserved areas, enhancing communication strategies, and fostering collaborations with local governments and health agencies can significantly improve the perceived and actual benefits of mining operations. Additionally, adopting a robust impact assessment framework before initiating projects is essential to identify potential risks and develop strategies to mitigate them effectively. Such frameworks will ensure that mining activities support sustainable development goals while minimizing harm to vulnerable populations.

This study acknowledges certain limitations, including its reliance on qualitative data and a focus on a single geographic area, which may limit the generalizability of the findings. Future research should consider comparative analyses that include control communities not directly impacted by mining activities. Such studies would offer a broader perspective on relative health outcomes and provide a stronger empirical foundation for policy recommendations. Longitudinal studies tracking changes over time would also be invaluable in understanding the long-term health impacts of mining operations. Additionally, exploring mixed-method approaches that combine qualitative and quantitative data would help triangulate findings and provide a more comprehensive understanding of mining’s health impacts.

Future studies should also assess the effectiveness of existing healthcare and social development initiatives implemented by mining companies to identify best practices and areas for improvement. Investigating the role of community engagement in shaping perceptions and outcomes would add another layer of understanding to the dynamics between mining operations and local populations. Furthermore, comparative studies across different mining regions, both within the Philippines and globally, would highlight contextual similarities and differences, allowing for the adaptation of successful interventions across diverse settings. Finally, incorporating advanced environmental monitoring technologies, such as remote sensing and real-time air and water quality data, could provide more precise assessments of environmental health risks and their impacts on local communities.

In conclusion, the findings emphasize the necessity for mining companies to adopt a comprehensive and inclusive approach to community health and environmental management. By proactively addressing health concerns, engaging stakeholders, and implementing sustainable practices, mining operations can balance economic growth with the well-being of the communities they affect, setting a model for responsible and equitable resource extraction. This study’s theoretical and empirical contributions underscore the importance of integrating community perspectives into decision-making processes to ensure that the benefits of mining extend equitably to all members of the community, while also advancing the broader understanding of how localized contexts influence the health impacts of industrial activities.

## Figures and Tables

**Table 1:** Summary of respondent population and demographics per study site.

Sex	Study Site					Total
	Malinao	Navarro	General Aguinaldo	San Antonio ^1^	Mining Site	
Male	3	3	3	7	3	19
Female	7	6	5	10	8	36
Total	10	9	8	17	11	55

^1^ San Antonio comprises the Sitio Balite and Sitio Melody communities.

## Data Availability

Data from this research are available from the authors upon request.
